# New Method of Constructing Full Dentures

**Published:** 1916-05

**Authors:** Clyde Davis

**Affiliations:** Lincoln, Neb.


### NEW METHOD OF CONSTRUCTING FULL DENTURES.

BY CLYDE DAVIS, B.S., M.D., D.D.S., LINCOLN, NEB.

I have a method which I wish to present to the dental profession for its consideration; a method of constructing a full denture, either single or double, that I have never read of, and I believe the idea or the application of the idea is original. I have tried it out in our infirmary, and proven that it is possible to get suction for all plates, both upper and lower, in every instance where the mouth approaches normal.

Stated succinctly, the method involves the complete abandonment of the model of the mouth and the plate is vulcanized on a cast which is the negative of an adhering base plate. Stated as briefly as possible, the method is as follows: An impression is taken of the mouth and the cast made, which should be somewhere near correct, but it is not necessary to take any great pains with it. It should give the outline of the completed plate and partially represent the conditions of the mouth. In fact, in some instances, even this is not necessary, provided you have a dummy cast of plaster, metal or celluloid which is somewhere near the size of the mouth, but which should be a trifle larger in every direction.

Having secured an approximately correct model, a specially prepared base plate, which is very thin and when warm is very pliable, is readily shaped over this model. This base plate will probably not show suction when placed in the mouth. This should then be trimmed to the proper size around the edges, placed in the mouth and pressed to position. If it is far wrong, take it out, warm it over a flame and press to place in the mouth. As soon as a little suction begins to show up, instruct the patient to keep the mouth closed and suck the plate as tightly as possible. The warmth of the mouth will change this until close adaptation results and strong suction will appear. This may be assisted by manipulating with the fingers and is particularly necessary with the trial base plate on the lower jaw. When this suction has been secured it will be found that this trial plate will not fit the original cast, even though it is a perfect model of the mouth. It will be found that no impression of the mouth can be taken which this adhering base plate will fit, which proves my contention for the past twenty years, that a plate made from a perfect cast of the mouth seldom has adhesion. I have always spoiled my plaster casts up to this time in order to get adhesion. Now I am asking you to throw them away altogether.

Coming back to the case where you have one or both adhering trial base plates in the mouth, I then take the bite in the usual way for that kind of a case using the built-up bite on these base plates. Plaster of Paris is then mixed up ready for use and the trial plates hastily removed from the mouth and filled with plaster. These are then mounted on the articulator and the case proceeds in the usual way. In these casts which are poured up you have the negative of an adhering base plate which is by no means a model of the mouth. Every plate made by this method, both upper and lower, adheres as would the trial base plates.

This method of making an adhering denture without vulcanizing on a cast of the mouth has many side issues which I have not time to go into at present. Suffice it to say that by this method we can construct a denture if necessary without having taken an impression of the mouth, using an approximate dummy to start the shape of the base plate, completing its form in the mouth assisted by the body temperature, which must slightly affect the specially prepared base plate. The plaster which is poured into these adhering base plates should be a little below body temperature in order that they may not give and change shape under the weight of the poured-in model. The base plates should be given a thin coat of quickly drying ether varnish before pouring the plaster, and then painted over with a very thin solution of soap. This gives a smooth cast, and the base plate by warming can be easily removed from the cast. This matter can be tested out by anyone. If he will try the base plate back in the mouth after it has been removed from this cast, it will be found that the adhesion is still there. If he will take the base plate and warm it and place it over the original cast of the mouth and press it into place, then try it in the mouth, it will be found that the adhesion has been destroyed. Allow this base plate to remain in the mouth and get warm and adhesion again takes place. Taking it out of the mouth it will be found to fit the cast which has been poured into the base plate and does not fit the cast of the mouth.—*Items of Interest.*

COMMENT.—This method of adopting artificial dentures strikes us as most unscientific and likely to be disastrous if practiced to any considerable extent. We should not reprint it except that its author is so well known and a most excellent teacher and professional man. Plates so made may have adhesion but they certainly will not displace soft structures or make for durability and sanitary oral conditions. If adopted at all use with caution and discretion. The method suggests an idea that may be practicable of determining the limits of plate margins, by means of wax base plates made on good models which may be worn by the patient long enough to establish tolerable extensions of the plate.—ED., REGISTER.

